# An Essay on Laborious Parturition: In Which the Division of the Symphysis Pubis Is Particularly Considered

**Published:** 1783

**Authors:** 


II. *An Effay on Laborious Parturition: in which the divifion of the Symphyfis Pubis is particularly confidered. By* William Ofborn, M. D. *Phyfician and Man-midwife to the General Lying-in Hofpital, in Store-ftreet, and Lecturer on Midwifery in London,* 8vo. Cadell, *London.* 1783. 271 pages.

THE operation, which is the chief fubject of this effay, has never been performed in this country, though on the event of a fingle cafe, the fuccefs of which appears to have been very problematical, it was recommended in the higheft terms of applaufe by the college of phyficians at Paris. If the advantages to be derived from the operation are really fo important as we have been taught to believe, it was incumbent on the practitioners of midwifery in *Britain*, to affign their reafons why they did not adopt the practice. If there was reafon to prefume that the operation had been unneceffarily performed, or that it was inferior in real utility to thofe those before practised; or if there were any proofs that false conclusions had been made from cases unfairly stated, those reasons and proofs should be rendered as public as possible, to deter the enterprizing from an operation which was so far from deserving commendation, that, if admitted into common practice, we had to dread the most deplorable consequences from it. With both these views Dr. Osborn has with great ability and candour given a full state of all that has been said upon this subject, and has proved that the operation is not only useless but pernicious.

The introduction our author employs, in proving the necessity of establishing midwifery as an art, from the peculiarities of the human construction. He rescues that branch of the profession from the severe and unmerited censures which witty, designing, or uninstructed men have endeavoured to cast upon it. These censures he proves to be groundless, and that the art of midwifery is equally necessary and useful with any other part of physic. This subject is managed with great strength of reasoning and accuracy of judgment, but we are of opinion, that the consideration of some other processes ceſſes of the conſtitution, the irregularity of or inability to perform which are the objects of medical attention, would have afforded a very good illuſtration, if not a powerful argument in favour of our author's poſition. With the intereſted diſputes of the profeſſion, ſociety is not concerned, but it ſeems to be allowed, that either no aſſiſtance is required at the time of parturition, or that aſſiſtance ſhould be given with ſkill and judgment. We likewiſe underſtand in the introduction, that the author means to confine his obſervations to that kind of laborious parturition, which is occaſioned by the diſtortion of the *pelvis*, and to that degree which has been lately ſuppoſed to require the ſection of the ſymphyſis of the oſſa pubis.

The work itſelf is divided into two chapters, and each of theſe into ſections. We ſhall have a perfect view of the manner in which the ſubject is conſidered by recapitulating the principal contents of each ſection, in a conciſe manner, as the nature of our work requires, yet ſo as to enable the reader to form his own opinion.

In the firſt ſection, the advantages ariſing from the incomplete oſſification of the head of the child, at the time of birth, are conſidered. This Dr. Oſborn ſuppoſes to be equal to all the difficulties which occur in natural parturition, and to thofe occafioned by leffer degrees of the diftortion of the pelvis. But, as he very juftly obferves, there is a fize below which the head cannot be diminifhed, and of courfe where this provifion may not be equal to the exigencies of a cafe. This leaft poffible fize he prefumes, and indeed proves, cannot be lefs than three inches in the fmalleft diameter of the head. If the dimenfions of the cavity of the pelvis are then reduced by diftortion below three inches, the head muft be leffened, or the woman muft die undelivered. To prevent the deftruction of the child, the Cæfarian operation has been practifed; and to preferve that, together with the life of the mother, the fection of the fymphyfis. The value of each of thefe operations is calculated from the event of the cafes, in which they have been performed, and the fuperior importance of the life of the mother to that of the child, is very fully and philofophically proved in the fecond fection. In this part of the enquiry, and in thefe calculations, the child is always prefumed to be living, when any of thefe operations are performed. But it could not efcape the fagacity of the author that his reafoning would have been ftrengthened, by obferving that that many of the children are dead when any of these operations can be propofed, and no perfon would recommend an operation, neceffarily fatal or extremely hazardous to the mother, without any profpect of preferving the life of the child.

In the third fection, the dangerous confequences of delaying to leffen the head, when the neceffity of doing it is clearly decided, are pointed out, as are the advantages of performing it early. In this fection Dr. Ofborn propofes, that in thefe cafes the head fhould be leffened, and that we fhould then wait for the expulfion of it, by the labour pains. Or if thefe are found infufficient for that purpofe, we are advifed to wait thirty hours, after the head is leffened, if the circumftances of the cafe will allow the delay, as the putrefaction of the child will render the extraction by far lefs difficult. This, which feems to be a great improvement in the operation, appears to be perfectly new; at leaft no author whom we recollect has mentioned it; yet the advantages which may refult from the flaccidity and foftnefs of the head, and other parts of the child, are obvious. In all cafes of this kind the author very wifely recommends a confultation, previous to the operation, and if this idea was to be extended, and a general rule made, that no operation in which the life of the mother and child are immediately concerned, ſhould ever be performed without a conſultation, the injunction would be very proper.

The fourth ſection contains an inquiry intended to determine the ſmalleſt dimenſions of the pelvis, through which it is poſſible to extract a child, when the head is leſſened. This, from a conſideration of the ſize of the baſe of the cranium, is decided to be one inch and a half. Hence it appears that in all caſes of diſtortion of the *pelvis*, in which the cavity is not reduced to leſs than three inches, we may hope that the child may be born by the force of the labour pains, or ſuch aſſiſtance as will not be neceſſarily injurious to the parent or child. When the ſpace is leſs than one inch and a half, the reduction of the ſize of the head would not anſwer the intention, as the child could not afterwards be extracted. The operation of leſſening the head of the child muſt therefore be confined to a limited degree of diſtortion, on one ſide of which it is not neceſſary, and on the other ſide uſeleſs. This explanation promiſes great advantage in practice, as the determination has hitherto been left, without any rule, to the judgment ment of every practitioner. All the preceding calculations feem to have been drawn from obfervations and reflections on the extremely curious cafe of Elizabeth Sherwood, a very unhealthy and deformed woman, not more than forty-two inches in height, who was delivered by the doctor with great fkill and dexterity, and who recovered without any untoward accident. As the hiftory of this cafe does not admit of abridgment, we mean to infert it at full length in fome future number of our Journal.

The fecond chapter begins with an account of the fection of the fymphyfis of the pubis. This was firft propofed and performed by M. Sigault, and approved and recommended by the college of phyficians at Paris. The applaufe which was given to M. Sigault was beyond all meafure precipitate and intemperate. A medal was ftruck to perpetuate his fame, and a penfion from his moft chriftian majefty was obtained for him. Nor was the fame of the operation confined to Paris or France, it fpread over the whole Continent. In Holland the operation was approved by the learned Camper; in Germany by profeffor Guerard of Duffeldorpe, and the prefident Siebold of Wurtzburg, and by many others. We have then a more particular account of of the means uſed to eſtabliſh the credit of the operation, which the royal academy of ſurgery, very much to the credit of that learned body, diſapproved and rejected in the firſt inſtance, as did Baudeloque, who examined its merit with great circumſpection and judgment.

Our author then enters upon a critical examination of the arguments and opinions of the favourers of the operation, particularly thoſe of Le Roy, Camper, Siebold, Guerard, Rouſſel de Vauzeſme, Loder, Benteley, Leake, and on all theſe our author animadverts, and oppoſes them, not with conjectures or preſumptive arguments, but with reaſons drawn from the event of the caſes recorded, many of which have been publiſhed, with a view of adding to the praiſe already beſtowed upon M. Sigault, and to ſupport that glory which the French nation muſt derive from the honour of giving him birth.

The reflections which our author makes upon this occaſion, ſhew him to be equal to the ſubject which he has undertaken to inveſtigate, and they are animated with the moſt lively principles of humanity. If the inferences which he makes are juſt, for the credit of the College of Phyſicians at Paris, it would be proper that the record cord of their proceedings in this bufinefs fhould be erazed, and their medals recalled.

Dr. Ofborn acknowledges, that, in this country, we are much indebted for the prevention of this operation to the early interference of the late Dr. Hunter. But although, in the publication alluded to, Dr. Hunter reprobates the fection as a fubftitute for the Cæfarian operation, and expreffes his doubts of its ever being of general ufe, and cautions againft its precipitate admiffion; yet he defcribes a fuppofed cafe where he thinks it might be a confiderable improvement in practice. This fuppofed cafe is, where, on account of the narrownefs of the pelvis, or partly from that circumftance and partly from a projection of the lumbar vertebræ, the child cannot be brought within the fphere of the crotchet, but where room fufficient for that purpofe may be gained by dividing the fymphyfis pubis. Dr. Ofborn doubts whether fuch a cafe ever did or can happen; and "when we advert," fays he, "to the maimed and weakened "ftate of the pelvis, and its confequent inability after the divifion of the fymphyfis, to fuftain the violence and repeated exertions, unavoidable in the ufe of the crotchet; and, at "the fame time, when we reflect upon the mif-

"chief,

"chief, that the soft parts must inevitably suf-

"fer from the division, particularly those which

"lie immediately behind, and in contact with

"the ossa pubis; first, by being torn from the

"bones to which they are naturally connected,

"afterwards, by being exposed for a consider-

"able time to the external air, and last of all,

"by being pressed against the divided edges of

"the bones, in the passage of the child's head;

"when, I say, all these circumstances are con-

"sidered, we must conclude, that the operation

"in the case supposed by Dr. Hunter, instead

"of '*giving the mother a good chance for life and*

"*tolerable health*,' will be as certainly fatal to

"her, as the crotchet must have already proved

"to the child."

In the conclusion of the work, the learned and experienced author takes the liberty of contradicting (not disrespectfully) some assertions of Professor Hamilton of Edinburgh, both concerning the event of the cases of the Cæsarian operation, and the event of the practice in the most deformed pelvis's which have occurred in London; and expresses his confidence in the Professor's candour and good sense to correct or retract his opinions, which Dr. Osborn thinks might otherwise be highly injurious to the interests of humanity.

